# Editorial: Genomic Insights and Translational Opportunities for Human Cancers

**DOI:** 10.3390/biomedicines14010111

**Published:** 2026-01-06

**Authors:** Apostolos Zaravinos, Ilias Georgakopoulos-Soares

**Affiliations:** 1 Cancer Genetics, Genomics and Systems Biology Laboratory, Basic and Translational Cancer Research Center (BTCRC), Nicosia 1516, Cyprus; 2 Department of Life Sciences, School of Sciences, European University Cyprus, Nicosia 1516, Cyprus; ilias@austin.utexas.edu; 3 Division of Pharmacology and Toxicology, Dell Paediatric Research Institute, College of Pharmacy, The University of Texas at Austin, Austin, TX 78723, USA

The focus of this Special Issue on Biomedicines is on genomic insights and translational opportunities for human cancers. Cancer is fundamentally a systems-level disease, and analyzing and integrating different modalities is necessary to further our understanding, identify new biomarkers, therapeutic vulnerabilities and treatments. While the traditional paradigm focuses on genetic mutations as the primary drivers of cancer initiation [1], contemporary evidence reveals an intricate network in which additional factors including epigenetic plasticity, metabolic reprogramming, and microenvironmental pressures interact with different types of genomic alterations to sustain tumor growth [2,3].

Large-scale cancer genome projects have elucidated the extensive genetic heterogeneity within and between tumor types [4]. Complementing this, analyses of chromatin modifications and DNA methylation patterns across cancer types have shown that epigenetic deregulation is central in shaping transcriptional states that underpin malignancy [5]. 

Various published studies echo this integrated view, reinforcing the concept that oncogenesis emerges from the collective behavior of multiple interlinked layers, including transcriptomic and metabolomic profiling and immunological programs, rather than from genetic mutations alone. Together, technologies to study these different layers form the foundation of modern precision oncology by providing a comprehensive map of cancer vulnerabilities that may be therapeutically exploited.

The Special Issue further emphasizes the critical influence of the tumor microenvironment (TME). Long considered passive structural context, the TME is now recognized as a highly dynamic compartment composed of a plethora of cell types including immune cells, fibroblasts, endothelial cells, and extracellular matrix components that actively shape tumor behavior [6,7,8]. The interplay between cancer cells and the TME is central to cancer development and progression. Targeting components of the TME also represents an opportunity for the development of more effective therapeutic strategies [6,7]. 

Although granzymes and perforin are central mediators of anti-tumor immunity, their expression patterns, genomic regulation, and alterations across cancer types have not been systematically characterized. Findings presented in this Special Issue address this gap through a pan-cancer genomic analysis, characterizing the roles of these cytotoxic effectors in tumor-immune interactions and evaluating their potential as biomarkers and therapeutic targets [9]. The findings of Mareboina et al. reveal that the distinct expression of *PRF1*, *GZMA*, *GZMB* and *GZMK* in pan-cancer, highlight their versatile roles in tumor immunity and play a critical role in shaping tumor behavior, disease progression, and therapeutic response [9].

In addition, Oketch et al. [10] compared the performance of different single-cell RNA sequencing (scRNA-seq) techniques to verify the concordance of the predictions based on biomarkers and those based on inferring copy number variations (CNVs) in pancreatic ductal adenocarcinoma. The authors compared the different tools among them in terms of their performance of inferring CNVs. They demonstrated that the predictions are strongly dependent on the sample and the software employed and cautioned on the reliability of the examined algorithms. The study by Oketch et al. reveals the need for more accurate and better tools to detect CNVs in scRNA-seq data [10].

Duodenal adenocarcinoma is a rare gastrointestinal malignancy, with limited molecular and genomic insight into the tumor microenvironment and its development. Huang et al. sought to discover new biomarkers for early detection and possible therapeutic targets in duodenal adenocarcinoma by analyzing differentially expressed long non-coding RNAs (lncRNAs). Their results indicate that the acidic microenvironment affects the disease phenotype through regulation of the Linc01559–GRSF1 axis [8].

Therapeutic resistance, whether inherent or acquired, remains a defining challenge in oncology, necessitating novel solutions. In addition to genetic mutations, resistance mechanisms often also involve epigenetic remodeling, metabolic rewiring, and microenvironmental interactions. Bonifacio-Mundaca et al. in their review [11] explore the interplay between metabolic and epigenetic mechanisms in medulloblastoma, with a focus on their functional roles and therapeutic implications. The authors conclude that metabolic–epigenetic crosstalk in medulloblastoma holds significant potential for the development of new therapeutic approaches. They also emphasize the importance of elucidating subtype-specific dependencies and integrating robust biomarkers for patient stratification, thereby enabling the development of precision medicine strategies that may improve clinical outcomes while minimizing long-term treatment-related toxicity [11].

Collectively, the studies collected in this Special Issue reflect the maturing landscape of cancer genomics, the integration of other omics data, and advances in translational oncology. The continued integration of genomic insights with epigenetic, metabolic, and microenvironmental perspectives promises to accelerate progress toward truly personalized cancer care, from the discovery of novel biomarkers to the development of more effective treatments.
